# Gravitating towards Fintech: A study on Undergraduates using extended UTAUT model

**DOI:** 10.1016/j.heliyon.2023.e20731

**Published:** 2023-10-06

**Authors:** Nahida Sultana, Rubaiyat Shaimom Chowdhury, Afruza Haque

**Affiliations:** a Department of Management Information Systems (MIS), Faculty of Business Studies, University of Dhaka, Dhaka, 1000, Bangladesh; b Department of Business Administration, Bangladesh University, Dhaka, 1207, Bangladesh; c Department of Humanities and Social Sciences, Dhaka University of Engineering & Technology (DUET), Gazipur, 1707, Bangladesh

**Keywords:** Fintech, UTAUT, Financial inclusion, SEM, Bangladesh

## Abstract

One of the most pressing issues affecting people worldwide is the accessibility of financial services in the wake of the COVID-19 outbreak. While peoples' adoption of technological advancements has spiraled over time, the acceptance of Fintech services edged up for instilling resilience during the pandemic. Hence, the study aims at exhuming the Fintech adoption for sustainable financial inclusion among young undergraduate users through the Unified Theory of Acceptance and Use of Technology (UTAUT) model. To test the model, the researchers obtained data from 375 undergrads. The study adopted the Structural Equation Modelling (SEM) approach in AMOS software to examine the determinants that affect the users to embrace Fintech services. The findings revealed that performance expectancy, effort expectancy, and facilitating conditions significantly influence the students' intention to use Fintech, whereas, the users’ facilitating condition and behavioral purposes positively influence them to use the financial technology. Interestingly, the social influence and personal innovativeness did not affect their intention to accept and adopt Fintech. Therefore, the study results will help explore the expectations, preferences, and actual level of use of Fintech among undergraduates and contribute to the escalation of Fintech use, leading to sustainable financial inclusion.

## 1 Introduction

The world is changing at a rapid pace. There has never been a time in history where nearly every area, including human life, economy, and politics, has been impacted by the rapid change brought about by advances in information technology [1]. The financial sector is one of the most significant stakeholders in this change through using technology, and it is so vibrant that we have a new jargon, "FinTech" (Finance + Technology). Despite its reputation as a "buzzword" in the media [2], argue that FinTech is deeply rooted in IT, innovation (tech hubs, investment in R&D, etc.), and the financial industry. Financial technology helps organizations, consumers, and owners better manage their financial tasks, such as operations, procedures, and lives, with specialized software programs and algorithms used on smartphones and computers in daily activities [3]. Our young generations will mainly use this change. Generation Z, or even the Alpha Generation, can do almost everything online, from taking pictures with their phones to buying things or sending money to other people. (Pintér et al., 2021). They will be the biggest customers of the technological changes in finance. As per 2022 data worldwide, 75 % of people aged between 15 and 24 are using the Internet [4]. In this paper, we have tried to understand how young graduates adopt FinTech, especially in Bangladesh.

The adoption of Fintech is still being determined [5], although numerous researchers and practitioners believe Fintech can transform the future of the financial industry [6]. argue fintech innovations such as e-financing and mobile technology have triggered a transition in the financial sector, which is now increasingly technology-driven and fraught with potential and challenges. Some users still need to be more hesitant to implement Fintech due to the substantial risks it poses. Though Fintech offers new opportunities to empower individuals by increasing transparency, decreasing costs or eliminating intermediaries, and making information accessible (Zuiderwijk, 2015), risk issues such as financial (e.g., loss of financial outcome and an additional fee), regulatory (e.g., legal uncertainty for adoption), security and privacy (e.g., the vulnerability of security technologies), and operational (e.g., inadequate processes or systems of Fintech companies) concerns are the most significant adoption barriers [5]. This paper also sought to collect the perspectives of recent Bangladeshi graduates on these issues.

We have seen the disruption caused by COVID-19 in our daily lives, affecting health, society, and the economy [7]. Then, we have observed how technology overcame the disruption to make our lives as smooth as before. Different technologies have different impacts. For example, some technology allows us to understand the COVID-19 impact on human psychology through applications [8]. On the other hand, some technologies like Computer-assisted audit tools and techniques (CAATTs) for audit have opened a new era of adaptation in post- COVID-19 [9]. Not only during COVID-19 or after, but technology is also the orchestra for changes almost everywhere. Enterprise information systems adaptation [10], De Lone and Mc Lean's Information System (D&M IS) used for sustainable AIS [11], digital accounting systems in improving the quality of decision-making in banks [12] are the new essentials just like the financial technology. Fintech is as vital as electricity for the financial sector.

Bangladesh herself is a fascinating field of study in the context of Fintech adoption among youths. There are nearly 58 million individuals in Bangladesh between the ages of 15 and 35, indicating a vast opportunity for technology-based businesses. Bangladesh ranks 77th in terms of availability and 86th in terms of affordability of financial services despite having nearly 2.2 % of the world's population. As a result, the percentage of people with a bank account needs improvement, with almost 70 % of the population needing one. Compared to the few people with bank accounts, over 117 million people have mobile subscriptions. As a result, in 2011, the government of Bangladesh established rules for Mobile Financial Services (MFS), and today, Bangladesh is home to more than 8 % of all mobile money accounts globally. Most of the deals are still about basic transactions. Therefore, MFS activities comprise the bulk of Bangladesh's Fintech ecosystem. However, other ecosystem services, including IT platforms, solution providers, and financial service providers, continue to employ MFS in conjunction with other businesses rather than maximizing the benefits of Fintech [13].

This research is dedicated to the broad understanding and acceptance of financial services among young local users in Bangladesh. Though there are several research works on Technology adaptation in Bangladesh, we have found that FinTech adaptation among young Bangladeshis is a remarkably untouched sector. Undergrad students are typically unable to access FinTech services because of financial and technological barriers. Therefore, a theoretical model has been suggested to examine the involvement and understanding of FinTech by Bangladeshi youths. In addition, this research aims to identify the theoretical aspects that can promote the knowledge and acceptability of FinTech services and products among the undergraduate students' sample size. There is also a discussion of how customers' attitudes and actions affect the adoption of FinTech offerings. Researchers, FinTech operators, policymakers, and enterprises could benefit from widespread recognition and adoption of FinTech goods and services. The study's findings are anticipated to be a game-changer for the digitalization of Bangladesh's economy.

This study employed the Unified Theory of Acceptance and Use of Technology (UTAUT) to determine the acceptance intentions of users towards Fintech services and products among Bangladeshi young undergraduates. We chose UTAUT mainly for its conceptual solid factor and expanded explanatory power [14,15]. It acknowledges that people's propensity to accept new technologies is influenced by more than just their views; it also depends on their social, organizational, and environmental contexts. Through UTAUT, this study examines the variables that influence the intention of Bangladeshi undergrads to utilize FinTech services and products. Performance Expectation (PE), Effort Expectation (EE), Social Factor (SF), Facilitating Condition (FC), Personal Innovativeness (PI), Behavioural Intention (BI), and Actual Use (AU) are the determining factors to evaluate the attitudes and intentions of Bangladeshi Graduate students about the use of FinTech services and products in this paper.

The following section provides a thorough introduction to the relevant literature, a description of the theoretical model, and some theories underpinning its development. To shed light on this topic, the UTAUT framework has been used to formulate a working hypothesis. The results and analyses of the data have been discussed in the subsequent section. Finally, the study concludes with a discussion of its shortcomings and some ideas for further investigation.

## 2 Literature review

### 2.1 Financial technology (fintech)

Researchers studied the relationship between technological advancement and financial transformation under various Fintech definitions. McKinnon and Shaw initially suggested the term "Financial Deepening" in 1973, which quickly advanced sci-tech in the international economy [16]. The monetary market has experienced a disruptive revolution due to the fusion of finance and information technology [17]. By bringing disruptive transformation in every aspect of financial services, Fintech is becoming important in the global economic system. The application of Financial Technology in the area of Inclusive finance for the betterment of society and the economy has become a common practice in India, China, and many other countries [18]. In today's competitive era, Fintech acts as a powerful weapon in the financial industry which has a tremendous impact on sustainable development [19]. [20] found in his study that Fintech positively impacts sustainable business performance [21]. also examined the role of Fintech as a mediator on the impact of business drivers on sustainable performance [21]. opined that Fintech has the potential to influence the business driving forces that facilitate the sustainable implementation of the Indonesian financial and banking sector. Bangladesh's current financial innovation scenario is good enough as the financial inclusion rate is higher than other developing countries [22]. From expanding branches to increasing profitability, innovation, competitiveness, and feasibility in the financial sector, Bangladesh faces outstanding progress with the help of different reform program applications and modern financial technology [23]. Mobile banking and agent banking significantly contribute to sustainable finance among the Fintech applications.

Mobile technology facilitates mobile banking, which tremendously impacts financial inclusion by providing sustainable financial services (Horne, 1985; [24]. Mobile banking has revolutionized the financial market by increasing the mobility of financial resources [25]. Fintech helps achieve sustainable finance and plays a pivotal role in ensuring financial inclusion. Financial inclusion is an invisible hand in empowering women and low-income people. Many studies examine the role of Fintech and financial inclusion in empowering women and people experiencing poverty. Still, only some of these studies are cross-country based and provide mixed findings. Very few studies examine the nexus of including Fintech and sustainable financial services in the context of developing countries, especially Bangladesh. Fintech facilitates financial inclusion, which ultimately ensures women's empowerment [26]. found in his study that financial inclusion helps improve women's income, purchasing power, standard of living, and family position. He added that rural women can now meet their needs and ensure better education and medical care for their children after being included in various financial inclusion programs. Financial service providers who struggle to serve people experiencing poverty with conventional economic production technology can now use innovative financial technology to provide financial services at a relatively low cost [25]. [18] opined that Fintech's advantages vary based on the financial sector. They also recommended that further exploration of Fintech's mechanisms, dynamics, and societal consequences will create much value.

### 2.2 Fintech among young population

Although to provide a note of comfort, debit cards continue to be Europe's preferred payment method across all age groups. Younger consumers who grew up after 1998 became full-fledged with the internet and digital technologies unquestionably prefer contactless and remote payments to the traditional use of cash or cards (Pintéret et al., 2021).

The adoption intention of Fintech is predominantly high among the young population. Some previous studies evidenced that young people use Fintech more than older people [27]. revealed in their descriptive findings that young people under 35 lead the experience of the experience of using Fintech. Mobile payments and digital wallets, as the latest trend, have been used unprecedently by young people like students. These practices are prevalent in some countries, such as India and China [28].

Pintéret et al. (2021) demonstrated that university students have the stable financial knowledge and indicated that the young generations assume a trade-off between financial literacy and digital attitude, where very few remark on the need for continuous development. Ahmad and Al Mamun (2020) provided insight into Islamic Fintech among young Muslim investors in Bangladesh. Bangladesh has large numbers of young males who can better use this technology. Several entrepreneurs in Bangladesh devise new ways to enhance financial markets through Fintech [29].

### 2.3 Fintech in Bangladesh

In Bangladesh, mobile banking is a widely used Fintech service among the people, especially young users. Azad (2016) coined the term mobile baking as the mobile financial services (MFS), and the adoption of MFS in the country began on March 31, 2011 (Azad, 2016; Khatun et al., 2021). Private commercial banks are expanding their mobile banking activities rapidly. Moreover, Bangladesh has made significant strides toward sustainable financial inclusion by establishing alternate distribution channels, such as electronic wallets, to provide low-cost financial services to the underprivileged and those in need [30]. [30] found that perceived risk, performance expectations, and effort expectations impact users' perceptions of the value of MFS platforms during the COVID-19 pandemic. In contrast, social factors, perceived trust, and perceived benefits strongly relate to users' intentions to adopt MFS platforms.

The status and the related works of other Fintech products and services are discussed as follows.

### 2.4 Theoretical and conceptual framework

#### 2.4.1 Extended UTAUT model

Information system research has uncovered a number of facets that influence technology adoption across a variety of acceptance models [31]. Among different technology adoption models the Theory of Reasoned Action (TRA) [32], the Theory of Planned Behavior (TPB) [33], the Technology Acceptance Model (TAM) [34], and the Unified Theory of Acceptance and Use of Technology (UTAUT) model [14] are significantly used. Whereas each of these models has benefits and drawbacks, UTAUT proved to be quite successful in evaluating the adoption of technology in various fields [14,31,[35], [36], [37]].

To adapt to the digital transformation of society, users are often forced to adopt and use new technologies. Researchers assert that UTAUT can account for up to 70 % of the variance in user intentions in numerous technology adoption studies [15,38]. According to Ref. [39]; UTAUT is an effective and widely used model to measure how users are adopting and using new technologies in both individual and organizational settings. UTAUT has acted as a paradigm for embracing diverse technologies in both corporate and individual circumstances [40]. Numerous studies [41,42] employed UTAUT model to predict the usage intention of Financial Technologies (FinTech) [43]. intend to investigate the elements that influence bank customers' acceptance of FinTech with the help of UTAUT model [44]. used UTAUT model in their study to examine the adoption of one of the important financial technologies, blockchain, in the banking sector and found that effort expectancy is a prime indicator of blockchain adoption in Malaysia's banking sector. To identify the acceptance factors of FinTech in the banking system [45], incorporated UTAUT and TOE theory in their study. They found that the most crucial elements for blockchain adoption in the Malaysian baking system are effort expectation, social influence, and facilitation. Subsequently, the basic UTAUT model underwent numerous modifications with the addition of different contextual and attitudinal variables [15,46,47] to get more concrete and useful results [47]. opined that depending on the context and goals of the study, researchers should either include or exclude the basic and extended UTAUT model elements. Recently, many academics have expanded the UTAUT model by including numerous new structures depending on various applications [31,48].

To examine the elements that influence people's acceptance of FinTech services, particularly on electronic wealth administration platforms [49], extended the unified theory of acceptance and use of technology (UTAUT) model by adding perceived risk (PR) and perceived value (PV) [50]. extend the UTAUT model by adding Perceived Risk (PR) and Perceived Trust (PT) to demonstrate the acceptance of mobile banking services among young Indian Users. The findings show that perceived risk, along with perceived trust, significantly impacts the connection underlying behavioral intention and actual use of mobile banking.

To achieve the study objectives outlined in the preceding section, this research extended Unified Theory of Acceptance and Use of Technology (UTAUT) model which is considered the most influential usage analysis and user acceptance model. Technology Acceptance Model (TAM) is the first and most commonly acceptable model for predicting users’ intention of adopting technology [51]. However [52], found in his study that the TAM has numerous shortcomings, particularly in its ignorance of social influence components of technology acceptance.

To overcome these shortcomings [14], constructed a model, namely “Unified Theory of Acceptance and Use of Technology (UTAUT)”, for investigating Information Technology-related adoption, especially in corporate settings [53]. The UTAUT model combines eight theories: Theory of Reasoned Action (TRA), Technology Acceptance Model (TAM), Motivational Model (MM), Theory of Planned Behaviour (TPB), Model Combining the Technology Acceptance Model and Theory of Planned Behaviour (*C*-TAM-TPB), Model of PC Utilisation (MPCU), and Innovation Diffusion Theory (IDT), all of which are concerned with the usage behaviour of Information Systems [14]. The basis of this research is The Unified Theory of Acceptance and Use of Technology (UTAUT) model. To conduct this study, we have adopted four determinants from UTAUT model: Performance Expectancy (PE), Effort Expectancy (EE), Social Influence (SI), and Facilitating Conditions (FC). This study extends this model with another determinant, namely Personnel Innovativeness (PI), to investigate the dominant factors that affect users' intention to utilize and adopt Fintech services. Researchers assert that UTAUT can account up to 70 % of the variance in user intentions in numerous technology adoption studies [15,38].

The UTAUT model, in conjunction with the expanded valence framework, is more suitable for adopting FinTech since it provides a deeper understanding of more contextual aspects [54]. That's why this study employed the extended UTAUT to determine the user acceptance intentions towards fintech services and products among young undergraduates of Bangladesh. To conduct the study, we have adopted four determinants from UTAUT model: Performance Expectancy (PE), Effort Expectancy (EE), Social Influence (SI), and Facilitating Conditions (FC). For predicting usage intentions toward Fintech and getting more useable outcomes, this study extended the basic UTAUT model by incorporating a contextual variable namely Personal Innovativeness (PE).

#### 2.4.2 Conceptual framework

Several numbers of research have been conducted on Fintech adoption, the perspective of customers on using it, and consumers' behavioural attitudes toward using different Fintech products in Bangladesh (Table 1). Yet, a gap is found in the perspective of the young generation of Bangladesh to adopt Fintech. To bridge the gap, the current study's motivation is to explore the behavioral intention and future prospects of Fintech solutions among the young cohort, specifically the undergraduates. Hence for this purpose, the UTAUT model is extended and used to uncover the behavioural attitude and intention toward Fintech.

**Table 1:** Fintech services in Bangladesh.

Fintech services	Status	Discussion
Online Banking Digital banking	• Fully functional • Licensed and allowed	[55] revealed that customers are more likely to adopt online banking at higher levels of performance expectancy (PE), effort expectancy (EE), and social influence (SI). In contrast, their Behavioural Intention (BI) toward digital banking is negatively impacted by facilitating conditions (FC).
Agent banking	• Twenty-nine banks were engaged in agent banking operations as of March 2022 through 19,530 locations run by 14,166 agents [56]. • 14,300 agents as of June 2022 [57]	• [58] showed that the most significant positive factors that influence peoples' intention to branchless banking include agents' trustworthiness and normative structure. • In order to comprehend and evaluate the case of Bangladesh and discover the elements associated to the adoption of agent banking in contrast to traditional banking [59], used the social exchange theory (SET) as our theoretical framework.
Crowdsourcing Crowdfunding	• Limited access • Inflow allowed; outflow constrained [60]	According to Ref. [61]; performance expectancy, effort expectancy, social influence, facilitating conditions, and perceived trust all significantly affect start-up companies' behavioural intention to use crowdfunding. Trialability and perceived trust, on the other hand, were not identified as important factors.
Digital money Digital payment Mobile Payment Mobile banking	Licensed by several legislations [60].	[62] revealed that the behavioural attitude towards the acceptance of mobile banking in Bangladesh is significantly affected by usage, psychological, and shopping benefits.
Blockchain Cryptocurrency	Implementation is illegal now.	Although many researchers proposed framework to implement blockchain in different sectors, the major causes of ongoing difficulty of integrating blockchain broadly in Bangladesh include the government's inflexible policy, the dearth of qualified experts, and a lack of funding [63]
RegTech in financial service/Share Market	Not implemented yet.	Bangladesh Bank is in ongoing discussion with stakeholders to apply Regulatory Technologies (RegTech)

**Fig. 1:**
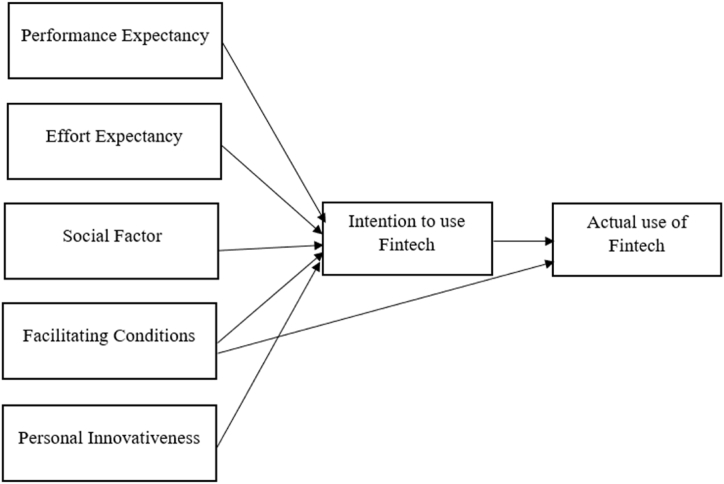
Conceptual Framework of the study.

The following conceptual framework (Fig. 1) has been formulated in this study keeping the determinants of UTAUT model. Here the model is extended by a variable ‘Personal Innovativeness’.

#### 2.4.3 Hypotheses development

Based on the conceptual framework shown in Fig. 1, several hypotheses have been formulated to bridge the gap found in previous works. The following segments discuss how each determinant of the extended UTAUT model supports the development of hypotheses.

##### 2.4.3.1 Performance expectancy (PE)

Performance expectancy (PE) refers to the user's belief that implementing a system will boost job performance [14]. According to Refs. [14,[64], [65], [66]]; PE is a potent tool that influences consumers' behavioural intention to adopt innovative and cutting-edge technology [67]. claim that PE has a significant impact on whether e-banking facilities are adopted. Hence, the supporting evidence supports the development of the following hypothesis.

H1 Performance expectancy positively impacts undergraduates' behavioural intention to use Fintech.

##### 2.4.3.2 Effort expectancy (EE)

The ease with which new technology can be used is described by effort expectancy [14]. EE has a significant impact on users' intentions to adopt new technology [68,69]; Hoque & Sorwar 2015). It significantly impacts the uptake of Fintech services, particularly e-banking solutions [67]. Therefore, the argument presented facilitates the development of the following hypothesis.

H2 Effort expectancy positively impacts undergraduates' behavioural intention to use Fintech.

##### 2.4.3.3 Social influence (SI)

Social Influence is a person ’s belief of how much important others perceive he or she must take on and employ modern technology [14]. According to Refs. [70,71]; SI has a significant impact on users' willingness to accept and implement a new system. Positive suggestions and peer endorsements encourage prospective customers to use Fintech services [72]. Previous research on the adoption and usage of m-banking and Fintech services has also found a link between social influence and the intention to utilize and embrace m-banking and Fintech services [[73], [74], [75], [76]]. Thus, the presented argument motivates the development of the following hypothesis.

H3 Social Influence has a positive impact on undergraduates' behavioural intention to use Fintech.

##### 2.4.3.4 Facilitating conditions (FC)

Facilitating Conditions (FC) refers to users' perceptions of how much a technical and organizational facilities can support the implementation of a new system [14]. According to Ref. [77]; FC plays a significant role in the adoption and use of new information and communication technologies. FC has a significant impact on Fintech application, particularly in online banking [78]. [79] evidenced that the Facilitating Condition (FC) significantly influence the consumers’ use behaviour of Fintech.

Consequently, based on the above arguments, hypotheses 4 and 5 of this research are as follows.

H4 Facilitating conditions have a positive impact on use undergraduates' behavioural intention to use Fintech.

H5 Facilitating conditions have a positive impact on users' actual use of Fintech.

##### 2.4.3.5 Personal innovativeness (PI)

Personal Innovativeness was added as a new attribute to the UTAUT model in this study (PI). The degree to which an individual is ahead of others in adopting a new technology or system is denoted by PI [80]. People who are more ingenious are more likely to experiment new systems or technology [81]. Multiple empirical studies have shown that employee innovativeness and behavioral intent to use technology are closely intertwined [82,83]. [27] revealed in their descriptive findings that young people under 35 years old lead the experience of using Fintech.

As a result, the argument presented encourages the establishment of the following hypothesis.

H6 Personal innovativeness has a positive impact on users' behavioural intention to use Fintech.

##### 2.4.3.6 Behavioural intention (BI)

Behavioural Intention (BI) describes a user's willingness to engage in or carry out a particular behavior [84]. BI is regarded as a reliable indicator of actual Use Behaviour (UB) in various research contexts [85,86]. According to Ref. [14]; BI can demonstrate how a technology or system is actually used. Since the introduction of Fintech and its integration into the founded financial services sector, behavioral intention has been viewed as an effective indicator of the likelihood of users' using and adopting Fintech services [87]. Hence, the argument presented supports the formulation of the following hypothesis.

H7 Users' behavioral intention has a positive impact on users' actual use of Fintech.

## 3 Methodology

### 3.1 Measurement items

The measuring constructs for latent components in the proposed framework were built from earlier works to support the validity of the measures. Each item's specific components and their associated sources are specified in Table 2.

**Table 2:** Measurement constructs.

Constructs	Constructing items	Sources
Performance Expectancy (PE)	PE1: I would find the Fintech services useful in my job. PE2: Fintech services enable to accomplish tasks more quickly PE3: Fintech services can improve my productivity PE4: Overall, I find Fintech services beneficial in my daily life	[15,88]
Effort Expectancy (EE)	EE1: It is convenient to use Fintech services EE2: Using Fintech is clear and comprehensible EE3: It is convenient for me to become skilful at Fintech services	[15]
Social Factor (SF)	SF1: People who impact on my behaviour think that I should use Fintech SF2: I'm more likely to use Fintech if my friends and family use it SF3: I use Fintech because my teachers use it	[88]; [14]
Facilitating Condition (FC)	FC1: I have knowledge of using Fintech FC2: I have resources (e.g. Smartphone) to use Fintech FC3: Fintech services are compatible with other system I use FC4: If I face any problem in using Fintech services, I can solve quickly	[89]; [15]
Personal Innovativeness (PI)	PI1: If I know any new technology, I look for ways to try out it. PI2: Among my peers, I am generally the first to experiment new technologies. PI3: Overall, I am not hesitant to experiment new technologies.	[90,91]
Behavioural Intention (BI)	BI1: I plan to use Fintech in future BI2: I intend to continue to use Fintech frequently BI3: I am used to Fintech Services	[15]
Actual Use (AU)	AU1: Fintech service is a pleasant experience AU2: I am using Fintech AU3: Use of Fintech is a good idea	[92]

### 3.2 Research paradigm and questionnaire design

The positivist paradigm, which holds that knowledge is produced by empirical observation, has been used as the study is quantitative [93]. Data are gathered using questionnaire surveys given to participants who use Fintech services for various reasons, and statistical measurements are used for data analysis in this deductive-level research project [93]. For collecting principal data from participants, a structured questionnaire was developed. The questionnaire contained three major parts and information about respondents’ summaries. Participants' summary includes information about gender and age. The next segment included a statement about the Fintech services they are using. The following section of the questionnaire contains UTAUT model constructs, where twenty-three words are included for seven research constructs. To boost response rate and quality with a focus on lowering respondents' levels of annoyance, all questions are based on a 5-point Likert-type scale [93] ranging from (1) Strongly Agree to (5) Strongly Disagree.

### 3.3 Respondents' consent and ethical endorsement

Under institutional policies and national law, social science research is not required to be approved in Bangladesh. Ethical review is only necessary for delicate scientific and medical experiments, such as animal trials. However, participation in the study was voluntary, and the author obtained the participants' approval before sharing their information with the public.

### 3.4 Population, sample and data collection

The target population of this research is the young undergraduates of different universities of Bangladesh. As the usage of financial technology among young people is more predominant (Pintéret et al., 2021; Pintéret et al., 2021; [28,29,94], this study focused on university students mainly. New Age Bangladesh (2023) published a report that, according to the University Grants Commission, the total number of students in 158 universities is 44,41,717 as of 2021.

Among these large young populations, samples are conveniently selected – where ten universities are public, and ten universities are private. Five hundred questionnaires have been disseminated online to collect data from the selection. The sampling procedure is convenient random sampling, where participants have been selected conveniently at random. This sampling strategy is used for easy access to the respondents. Four hundred three questionnaires have been returned from the respondents from 500 disseminated questionnaires. Three hundred seventy-five questionnaires were found occupied and correct; the rest were not found valid. Therefore, the present study finalized the total sample size of 375, representing a 75 % response rate, for further statistical treatment. Data collection has been completed from May 2022 to July 2022.

### 3.5 Statistical treatment of data

For analyzing data, collected responses were arranged and coded in SPSS version 28. The maximum likelihood technique – a statistical approach based on Structural Equation Modelling (SEM) was adopted to test and validate the research constructs. This study uses Structural Equation Modeling (SEM) to analyze data. Structural Equation Modeling (SEM) is a statistical tool that combines regression with confirmatory factor analysis [95]. The literature contains a significant range of viewpoints on choosing the right sample size for different kinds of statistical analysis [96]. For instance, for statistical analysis utilizing Structural Equation Modelling (SEM), a sample size of 200 is considered reasonable and 300 as good [97]. Similar to this [96], recommended using 200 samples to evaluate a model using SEM. According to Ref. [98]; a sample size of 200 should be utilized in any estimating technique to get reliable results. The sample size should be minimum ten times as large as the number of study items to be able to perform multivariate research, including multiple regression analysis [99]. This model is considered a standard tool for providing reliable results for a sample size of 200 or more [100]. and Kline (2010) recommended that 100–200 should be the minimum sample size for applying SEM. The current research includes 23 constructs; therefore, the sample size of 375 is much more appropriate to evaluate through SEM based on previous studies. The SEM model is used extensively in analyzing various phenomena and processes of sociology and social policy [101]. This model was also applied in various marketing and economics research [[102], [103], [104]]. This study focuses on measuring users' intention toward FinTech in the context of Bangladesh, which has a significant implication on social and economic policy making. For all these reasons, the authors employed SEM in this study. The extended UTAUT model was drawn using SPSS AMOS to validate the significance level of the variables.

## 4 Data analysis

### 4.1 Demographic profile

**Fig. 2:**
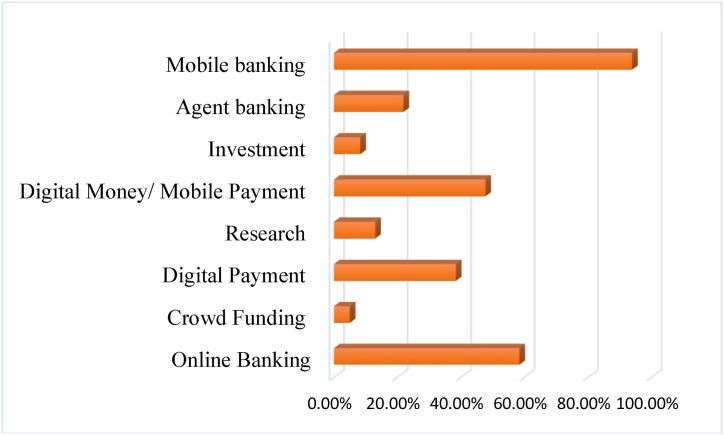
Usage of Fintech services.

Participants’ demographic features are listed in Table 3. For the studies examined, 273 (72.8 %) were males and 102 (27.2 %) were females. The majority of the undergraduates (72 %) were aged between 20 and 25 years. In addition, usage of Fintech services was analyzed, where respondents were open to pick more than one options given in questionnaire. It presents (Fig. 2) that most of the participants use Fintech services in the form of mobile banking (93.9 %), followed by online banking (58.4 %), and mobile payment (47.7 %).

**Table 3:** Demographic details.

Variables	Description	Frequency	Percentage
Gender	Male	273	72.8 %
	Female	102	27.2 %
Age	20–25	275	74.33 %
	26–30	95	25.37 %
Usage of Fintech services (More than one option was open)	Online Banking	219	58.4 %
	Crowd Funding	19	5 %
	Digital Payment	144	38.4 %
	Research	49	13 %
	Digital Money/Mobile Payment	179	47.7 %
	Investment	31	8.3 %
	Agent banking	82	21.9 %
	Mobile banking	352	93.9 %

### 4.2 Data reliability testing

The consistency of the research instrument evaluates the internal reliability of the items. The reliability test demonstrates how error-free the research tools are. Cronbach's Alpha (α) is an accepted indicator of construct internal reliability and scale consistency. According to Ref. [105]; a Cronbach's Alpha coefficient value above 0.70 indicates that a research construct is reliable and internally reliable.

As shown in Table 4, the Cronbach's Alpha for the identified 23 constructs of Perceived Efficiency (PE), Effort Efficiency (EE), Social Factor (SF), Facilitating Condition (FC), Personal Innovativeness (PI), Behavioural Intention (BI), and Actual Use (AU) are well above 0.70. Therefore, it presents strong evidence for the consistency of the constructs. Moreover, the ranges of Cronbach's Alpha if Item Deleted are specified below; those shows strong internal consistency as well. In Social Factor (SF) construct, there were 4 items originally. After removing one item that was inconsistent, it resulted in an increase in Cronbach's Alpha from 0.699 to 0.730.

**Table 4:** Reliability test of the research constructs.

Constructs	No of items	Cronbach's Alpha	Range of Cronbach's Alpha if Item Deleted	Range of corrected item-total correlation
**Perceived Efficiency (PE)**	04	0.778	0.706–0.764	0.507–0.633
**Effort Efficiency (EE)**	03	0.728	0.701–0.710	0.493–0.602
**Social Factor (SF)**	03	0.730	0.700–0.737	0.523–0.543
**Facilitating Condition (FC)**	04	0.739	0.710–0.736	0.447–0.607
**Personal Innovativeness (PI)**	03	0.747	0.714–0.717	0.531–0.627
**Behavioural Intention (BI)**	03	0.814	0.780–0.796	0.613–0.707
**Actual Use (AU)**	03	0.737	0.716–0.727	0.515–0.620

### 4.3 Model fit summary

The model fit measures are specified in Table 5. This presents the indices of appropriate measures and compares adequate fit value and present index value. The last column of the table illustrates the derived fitness of indices. Among the index values, most of the values are derived with adequate fitness.

**Table 5:** Research model fit summary.

Name of fit measures	Indices	Acceptable fit	Present Index Value	Derived fitness
**Absolute fit measures**	CMIN/Df	Lower than 5	2.445	Fit
	GFI	Higher than 0.90	0.906	Fit
	AGFI	Higher than 0.90	0. 858	Not fit
	RMSEA	Lower than 0.10	0.062	Fit
**Incremental fit measures**	NFI	Higher than 0.90	0.863	Not fit
	CFI	Higher than 0.90	0.913	Fit
	TLI	Higher than 0.90	0.910	Fit
	IFI	Higher than 0.90	0.914	Fit
**Parsimonious fit measures**	PGFI	Higher than 0.50	0.850	Fit
	PCFI	Higher than 0.50	0.776	Fit
	PNFI	Higher than 0.50	0.733	Fit

### 4.4 Path illustration of Structural Equation Modelling

**Fig. 3:**
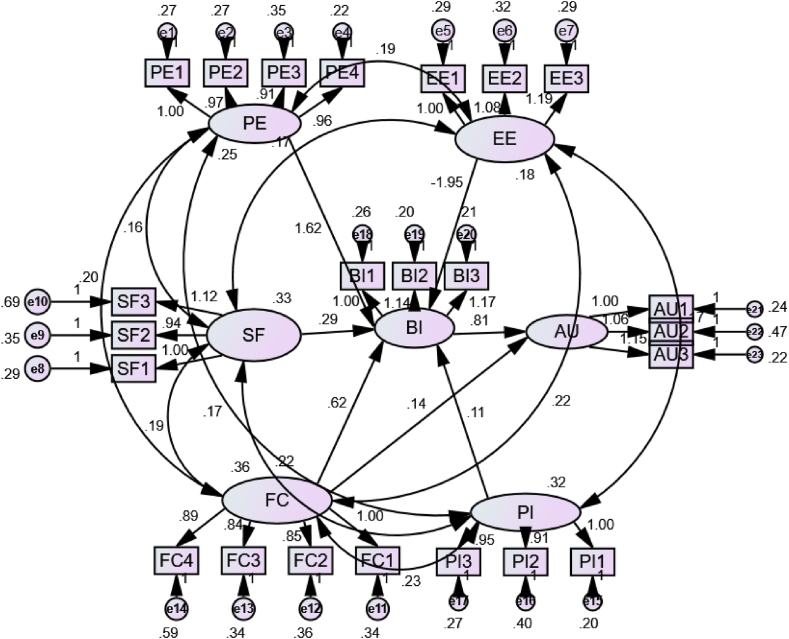
Final Path Diagram for SEM using AMOS.

The factor loadings of all variables are presented in Fig. 3. Factor loading values well above 0.40 are moderately significant, and 0.50 are extremely significant [106]. In the path diagram, the factors’ values considered include the values of Performance Expectancy (PE), Effort Expectancy (EE), Social Factor (SF), Facilitating Condition (FC), Personal Innovativeness (PI), Behavioural Intention (BI), and Actual Use (AU), for all the factors which influence PE, i.e., PE1 (1.00), PE2 (0.97), PE3 (0.91), and PE4 (0.96) are more significant than the absolute values. Accordingly, all the variables are related to their role in determining the values of PE, EE, SF, FC, PI, BI, and AU values. Hence, all the factors specified in the framework for identifying the values of latent factors are relevant and highly significant.

### 4.5 Hypotheses testing

Table 6 deciphers the regression weights based on maximum likelihood estimates. The results present the paths of hypotheses, S.E., C.R., P- value, and comments. Here, S.E. shows the deviation derived in the computation of variables, and findings show that there are less variations in all derived S.E. values. The column P-value indicates that all the hypotheses, except H3 and H5, are accepted; for each variable, the significance value is higher than the acceptable level of the study, i.e., 0.05. This finding is further validated by the C.R. (z-score) value i.e. 5.386 for H1, 3.281 for H2, 2.733 for H4, 2.490 for H6, and 9.503 for H7 which much higher than the charted z-value of 1.96. Hence, for the current research, the examination of the undergraduates’ perception presents that Performance Expectancy (H1), Effort Expectancy (H2), and Facilitating Conditions (H4) have a significant positive influence on their Behavioural Intention as well as Facilitating Condition (H6) and Behavioural Intention (H7) have a significant impact on their Actual Usage.

**Table 6:** Regression weights.

Hypotheses	Path	S.E.	C.R. (z- value)	P Value	Comments on Hypotheses
H1	BI < ---PE	0.301	5.386	0.000	**Supported**
H2	BI < ---EE	0.595	3.281	0.001	**Supported**
H3	BI < ---SF	0.166	1.746	0.081	Not Supported
H4	BI < ---FC	0.228	2.733	0.006	**Supported**
H5	BI < ---PI	0.164	0.670	0.503	Not Supported
H6	AU < ---FC	0.057	2.490	0.013	**Supported**
H7	AU < ---BI	0.085	9.503	0.000	**Supported**

### 4.6 Undergraduates’ current and future level of fintech use

**Fig. 4:**
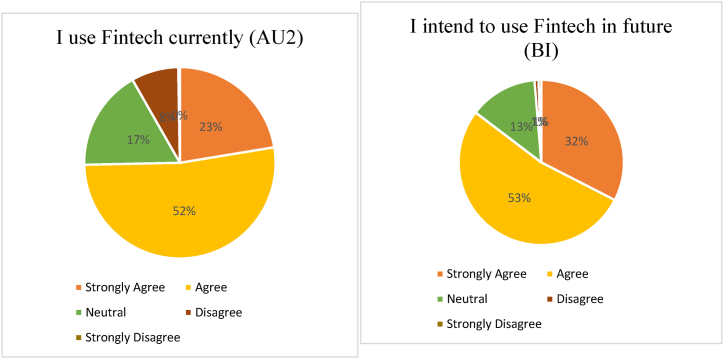
Current and Future Levels of use.

The current level of Fintech use among undergraduates has been specified in Table 7 and Fig. 4. The result reveals the frequency of current use (AU2) and intention to use in future (BI). Most of the respondents (52.3 %) use Fintech currently, and 52.8 % of the participants plan to use Fintech in future.

**Table 7:** Users’ current level of actual usage (AU2) and behavioral intention (B1).

	I use Fintech currently (AU2)		I intend to use Fintech in future (BI)	
	Frequency	Percentage	Frequency	Percentage
Strongly Agree	84	22.4 %	122	32.5 %
Agree	196	52.3 %	198	52.8 %
Neutral	64	17.1 %	50	13.3 %
Disagree	30	8.0 %	3	.8 %
Strongly Disagree	1	.3 %	2	.5 %

## 5 Discussion

The present research identifies the intention of undergraduates to use Fintech services using the widely used UTAUT model. The data are analyzed using AMOS structural equation modeling, and hypotheses are tested using regression weights. Before that, data reliability testing and study model fit indices are measured to evaluate data consistency and model fitness. Findings demonstrate the factor loadings of variables, hypotheses results, and descriptive figures of undergraduates' current and future levels of Fintech use.

Respondents' demographic profile shows that most participants are male and below 25 years old. Thus, the study presents the perceptions of the youngest cohort, where the attitude toward Fintech of the Male gender is dominant. Other descriptive figures show (Fig. 2) that the majority of undergraduates use mobile banking services (93.9 %), followed by online banking (58.4 %) and mobile payment (47.7 %). Fintech has become an alternative solution for financial transactions, especially for making payments. This is in line with the research from Ref. [28]; who demonstrated that this trend of Fintech solutions is predominantly high among young people. Very few young people use it for investment purposes and crowdfunding. As they are now merely undergraduates, this usage in investment and crowdfunding may rise when the undergraduates become entrepreneurs after graduation.

In terms of Fintech adoption intention, the study findings reveal that performance expectancy has a positive significant impact on undergraduates' Behavioural Intention. This result is consistent with the previous studies of Tohang and Anggraeni (2021) and [107] but contradicts with [108,109]. Besides, effort expectancy has a positive significant impact on undergraduates' Behavioural Intention which confirms the study conducted by Refs. [[108], [109], [110]]; however, opposed to Refs. [107,111]. Moreover, in the current study, Facilitating Condition is evidenced to impact undergraduates' Behavioural Intention positively. This finding favors the result found by Refs. [79,108,109,111]. Surprisingly, Social Factors do not have a direct positive significant impact on the behavioral intention of undergraduates. This result confirms the past finding of Tohang and Anggraeni (2021) and opposes [79,108,109,111]. Another remarkable thing is Personal Innovativeness has no significant impact on undergraduates' Behavioural Intention. Young generations are more interested in using newer technology; nevertheless, this factor is insignificant. This can be suggested as a mediating or external factor influencing the relationship between other factors and behavioral intention.

In terms of actual use of Fintech services, the current study evidenced that Facilitating Condition has a positive significant impact on undergraduates' Actual Use of Fintech services, and Behavioural Intention has a positive significant effect on undergraduates' Actual Use of Fintech services. This is in line with [108].

The above discussion shows the past findings that are consistent or contradictory with the current findings. However, the past studies are separate from the adoption intention of the young cohort of Bangladesh. This study attempted to bridge the gap, and based on the findings, the youngest peoples' behavioral intention is significantly influenced by their performance expectancy, effort expectancy, and facilitating condition, and their actual use is significantly influenced by the facilitating condition and their behavioral intention. Hence, the H1, H2, H4, H6 and H7 are supported.

Additionally, undergraduates' current and future Fintech use has been observed. Results indicate that over 50 % of the users are currently using Fintech services and intend to use Fintech products in the future. It is interesting to note that users who disagreed with the fact they are not presently using Fintech strongly agreed that they intend to use it in the future. The results suggest that the future of Fintech is more prospective among the youngest people of Bangladesh.

## 6 Conclusion

The world is developing at very fast pace with the technological advancement, especially after the outbreak of pandemic coronavirus. Bangladesh is changing to cope with the rise, where the growth of Fintech services is propelling the daily activities of general people from primary to tertiary level. Recent developments in Bangladesh's Fintech industry are significant, particularly in light of the new coronavirus outbreak. Nationwide, the financial activities of students are greatly influenced by the adoption of Fintech products and services. Fintech applications are used in almost all fields to carry out financial operations. This study aimed to identify the variables affecting undergraduates' inclination to use Fintech applications. This study used the UTAUT model to determine these parameters. The examination of structural equation modeling revealed that several factors affect students' attitudes to using Fintech products and services.

Performance expectancy of Fintech services positively impacts the undergraduates' intention to use Fintech service because the users find it useful, quick, and productive in their activities. In addition, effort expectancy showed a positive influence as the users get inspired by the convenience, clarity, and comprehensiveness of the service offered by Fintech products. Moreover, facilitating conditions positively impacted undergraduates' behavioral intention and actual use of Fintech services as the users possess adequate knowledge and resources to use the service. And finally, their behavioral intention toward using Fintech leads to their actual use of Fintech. However, the social factor doesn't impact their choice to use Fintech as the usage of Fintech among family members, friends, teachers, or people who motivate their behavior doesn't significantly influence their behavioral intention to use Fintech. As such, personal innovativeness does not impact undergrads' intention since the users' promptness to experiment with new technology doesn't influence their desire to practice financial technology.

The services of Fintech products, including blockchain and bitcoin, are expected to grow substantially in the future. Fintech service providers must work on facilitating the factors that influence their behavioral intention to use the actual use of Fintech products with a focus on the expectations and preferences of users from the services they demand.

### 6.1 Theoretical implication

This study contributes to the theoretical foundation that explores the Fintech adoption intention among the young population of Bangladesh. Moreover, the study employed the UTAUT model approach to facilitate the findings. However, very few studies identified the adoption intention of Fintech among undergraduates. Therefore, the results (H5, H6, and H7) will open a new avenue for future researchers. The findings of this study can further be used in exploring the expectations, preferences, and actual level of use of Fintech products among the targeted users.

### 6.2 Managerial contribution

In the epoch of Industry 4.0, digital transformation is no exception. Hence, the willingness of people to adopt the new technologies is a must to make the transformation possible. Significantly, young people's intention is important as they are more likely to experiment with new technology than other aged people. The study will contribute to understand intention, usage perspective, and the future prospects of Fintech adoption among the young cohort of Bangladesh. The study results are significant for Fintech service providers, scholars, researchers and designated government agencies. The enhanced advantages and necessary support services that are provided to users should be the Fintech service providers' main priorities. Additionally, sufficient advertising should be done to raise users' knowledge of products and services. Likewise, government agencies can boost the adoption intention and use of Fintech products and services among the people by mandating them to reimburse government-related payments through mobile Fintech, resulting in the increased adoption of Fintech services.

### 6.3 Limitations of the study

This study also has a few shortcomings that should be addressed. In this research, authors only looked at Bangladeshi undergraduate students; different findings may be revealed when considering secondary, postgraduate, or other level students here or in other developing nations. Additionally, the moderating impacts of users' demographic features, for example, age, gender, voluntariness, and experience were not examined in this research. For example, the mediating effects examined by Rahim et al. (2023) for islamic finance adoption could have been inspected in the current study for the young population in Bangladesh. Finally, while the overall effective sample size matches the minimal sample size proposed by Ref. [106]; it is still advised that future studies collect more data to increase the accuracy of the FinTech adoption model. All of these limitations open an avenue for performing future studies.

### 6.4 Future directions of the study

Future studies could consider the cross-cultural comparisons of the extended UTAUT model between different developing countries. Researchers can modify the research model outlined in this work to include various contextual factors, such as task technology fit, technology experience, etc. Different environmental elements, like the impact of government restrictions on the adoption of FinTech, may be the subject of future research.

## Funding statement

This research received no specific grant from funding agencies in the public, commercial, or not-for-profit sectors.

## Data availability statement

Data will be made available on request.

## CRediT authorship contribution statement

**Nahida Sultana:** Conceptualization, Formal analysis, Methodology, Software, Validation, Writing – original draft. **Rubaiyat Shaimom Chowdhury:** Investigation, Resources, Supervision, Writing – review & editing. **Afruza Haque:** Data curation, Investigation, Visualization, Writing – original draft, Writing – review & editing.

## Declaration of competing interest

The authors declare that they have no known competing financial interests or personal relationships that could have appeared to influence the work reported in this paper.
